# Satellite Tracking on the Flyways of Brown-Headed Gulls and Their Potential Role in the Spread of Highly Pathogenic Avian Influenza H5N1 Virus

**DOI:** 10.1371/journal.pone.0049939

**Published:** 2012-11-28

**Authors:** Parntep Ratanakorn, Anuwat Wiratsudakul, Witthawat Wiriyarat, Krairat Eiamampai, Adrian H. Farmer, Robert G. Webster, Kridsada Chaichoune, Sarin Suwanpakdee, Duangrat Pothieng, Pilaipan Puthavathana

**Affiliations:** 1 Faculty of Veterinary Science, Mahidol University, Nakhon Pathom, Thailand; 2 Department of National Park, Wildlife and Plant Conservation, Ministry of Natural Resource and Environment, Bangkok, Thailand; 3 Wild Ecological Solutions, Fort Collins, Colorado, United States of America; 4 Division of Virology, Department of Infectious Diseases, St. Jude Children Research Hospital, Memphis, Tennessee, United States of America; 5 Department of Microbiology, Faculty of Medicine Siriraj Hospital, Mahidol University, Bangkok, Thailand; The University of Hong Kong, China

## Abstract

Brown-headed gulls (*Larus brunnicephalus*), winter visitors of Thailand, were tracked by satellite telemetry during 2008–2011 for investigating their roles in the highly pathogenic avian influenza (HPAI) H5N1 virus spread. Eight gulls negative for influenza virus infection were marked with solar-powered satellite platform transmitters at Bang Poo study site in Samut Prakarn province, Thailand; their movements were monitored by the Argos satellite tracking system, and locations were mapped. Five gulls completed their migratory cycles, which spanned 7 countries (China, Bangladesh, India, Myanmar, Thailand, Cambodia, and Vietnam) affected by the HPAI H5N1 virus. Gulls migrated from their breeding grounds in China to stay overwinter in Thailand and Cambodia; while Bangladesh, India, Myanmar, and Vietnam were the places of stopovers during migration. Gulls traveled an average distance of about 2400 km between Thailand and China and spent 1–2 weeks on migration. Although AI surveillance among gulls was conducted at the study site, no AI virus was isolated and no H5N1 viral genome or specific antibody was detected in the 75 gulls tested, but 6.6% of blood samples were positive for pan-influenza A antibody. No AI outbreaks were reported in areas along flyways of gulls in Thailand during the study period. Distance and duration of migration, tolerability of the captive gulls to survive the HPAI H5N1 virus challenge and days at viral shedding after the virus challenging suggested that the Brown-headed gull could be a potential species for AI spread, especially among Southeast Asian countries, the epicenter of H5N1 AI outbreak.

## Introduction

To date, 17 hemagglutinin (HA) and nine neuraminidase (NA) subtypes of influenza A viruses have been identified. All the HA and NA subtypes have been isolated from wild aquatic birds, particularly from orders *Anseriformes* (ducks, geese, and swans) and *Charadriiformes* (gulls, terns, and shorebirds). Therefore, aquatic birds are widely accepted as the main natural reservoirs of influenza A viruses. Influenza viruses isolated from these birds are the mostly avirulent, low pathogenic avian influenza (LPAI) viruses [1]–[3].

Only some members of the H5 and H7 subtypes are highly pathogenic, but the H5N1 viruses were more virulent [1], [3]. In the first identified occurrence of H5N1 HPAI infection in humans in Hong Kong in 1997, 18 humans were infected and 6 died (case fatality rate 33.3%) [4]. The HPAI H5N1 virus re-emerged in Hong Kong in 2001, twice in 2002, and subsequently in 2003 in poultry, waterfowl and wild birds [5]. The viruses isolated in Hong Kong before 2002 were pathogenic in gallinaceous birds but not domestic or wild waterfowl. Death of aquatic birds from the HPAI H5N1 virus was first recognized in the 2002 outbreak [5]. The resurging HPAI H5N1 strain is highly virulent in both avian and humans, with a fatality rate of approximately 60% being reported in infected humans [6], [7]. In January 2004, an outbreak of H5N1 HPAI was reported in poultry and humans in Thailand. The virus initially isolated in Thailand belonged to clade 1; while clade 2.3.4 virus was introduced into northeast Thailand in 2006 [8], [9]. However, all of the virus isolates in central Thailand still remained in clade 1. There were 25 cases reported in humans, with 17 deaths (case fatality rate 68%). No cases of human infection have occurred since August 2006, but there were some avian influenza virus (AIV) outbreaks in poultry until 2008 [6], [7].

Based on virological data and satellite telemetry studies, many groups of investigators have linked migratory birds with H5N1 HPAI spread [10]–[17]. The first evidence that supported this claim was an outbreak of genotype z, clade 2 H5N1 viruses that occurred in wild bird populations at Qinghai Lake, western China during late April until June 2005 causing death of more than six thousand birds. The main victim infected were Bar-headed geese (*Anser indicus*) which accounted for more than 50% of the deaths, whereas Brown-headed gulls (*Larus brunnicephalus*, Family *Laridae*), were the second most species affected [10], [11]. Subsequently, H5N1 HPAI viruses similar to those in the Qinghai area were also isolated in other Asian countries, Europe, the Middle East and some African countries [2]. H5N1 viruses were likely transmitted along the flyways of birds that shared habitats, wintering sites, breeding areas or stopovers [2], [15]. In addition, the results from surveillance for H5N1 HPAI in migratory and wildlife birds in 14 provinces of China from 2004 to 2007 showed that highest infection rates (4.37%) occurred in mallards; while it was 2.39% for Brown-headed gulls [18]. Birds in the Qinghai province had the highest rates of infection, possibly because there are many lake areas in Qinghai, which are natural habitats and breeding ground of numerous bird species [11], [18]. Therefore, AI spread between birds sharing the same water rich habitats is more common. Investigation of the outbreaks and phylogenetic analysis suggested that the migrating birds in Qinghai got infected with H5N1 HPAI viruses through transmission from domestic poultry [11].

Satellite telemetry has been conducted by several groups of investigators to study the potential role of migrants in H5N1 HPAI spread [14]–[17]. With this technique, flyways of migrants can be illustrated with high accuracy; and the linkage between bird locations and geographical areas of AI outbreaks can be determined. Spatial analysis of distance of migration, duration of asymptomatic infection and duration of viral shedding strongly supported potential role of migratory birds on H5N1 HPAI spread. However, those satellite telemetry studies explored the flyways which spanned Africa, Europe and East Asia, while none involved flyways to Southeast Asia which was the epicenter of the outbreaks.

**Figure 1:**
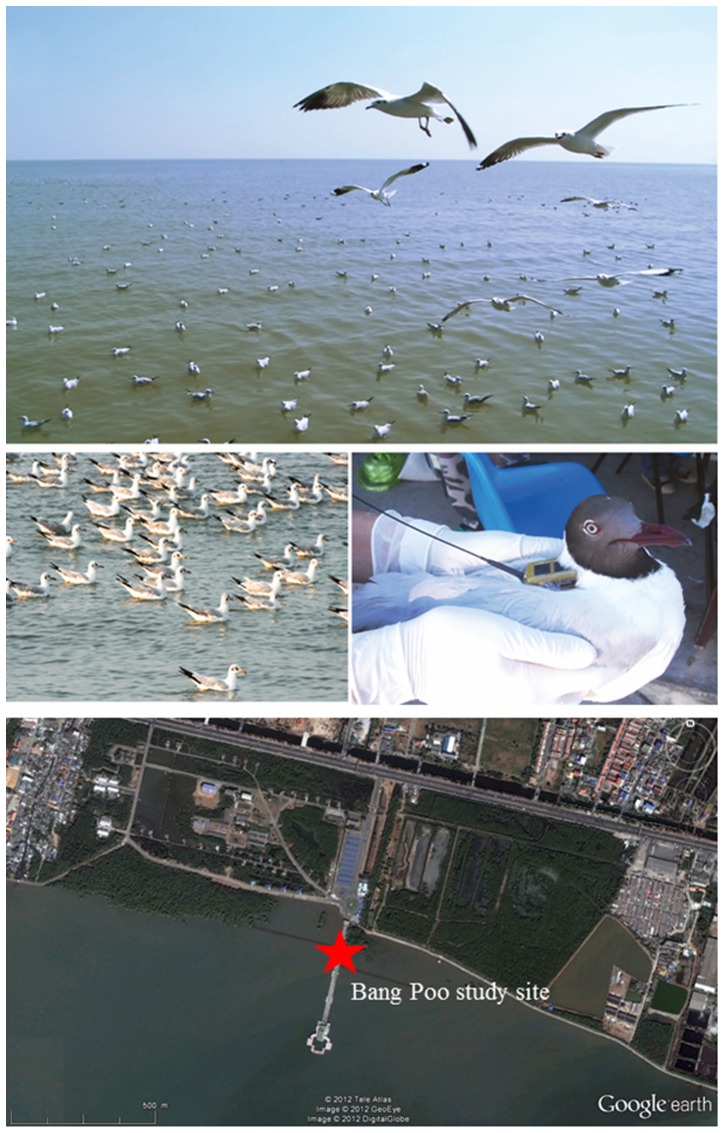
Flock of Brown-headed gulls and a gull fitted with satellite transmitter at the Bang Poo study site.

The Brown-headed gull is one of the most common bird species to annually visit Thailand, staying from October to May [19]–[22]. According to the Department of National Parks, Wildlife and Plant Conservation of Thailand, there are 2 large flocks of Brown-headed gulls in Thailand. The larger flock consists of approximately 5,000 birds foraging around the inner gulf of Thailand mainly at mangrove mudflats in the Bang Poo Rest and Rehabilitation Center of the Royal Thai Army, Samut Prakan province (Figure 1). The Department of Park, Wildlife and Plant Conservation by support from our group at the Faculty of Veterinary Science, Mahidol University isolated HPAI H5N1 virus from 1 of 23 gulls from this flock in 2005, none of 47 gulls in 2006 and 2007 and 3 of 83 gulls in 2008. Collectively, 4 of 153 (2.6%) apparently healthy birds in this flock harbored HPAI H5N1 virus infection. This data had been reported in Thai in the Wildlife yearbook, Department of Park, Wildlife and Plant Conservation. Source and place of infection in this flock of gulls is not known; nevertheless, the infected, apparently healthy gulls may further spread the HPAI virus along its flyways. However, the entire migratory route of Brown-headed gulls remains unknown. In order to determine the potential role of this bird species on AI spread along its migratory path, satellite telemetry was employed to track the flyways of Brown-headed gulls during 2008–2011. We searched for linkage between bird locations and occurrence of reported H5N1 HPAI outbreak while the birds overwintered in Thailand. Status of H5N1 HPAI infection in the flock was also determined. Gulls in captivity were challenged with H5N1 HPAI virus. Duration of viral shedding, tolerability of gulls to survive the infection till death together with the data on the distance and time spent on migration were explored in order to investigate the supportive role of gulls on AI spread along their migratory routes.

## Results

### Migratory routes and flight distances of Brown-headed gulls

Eight Brown-headed gulls employed in the satellite telemetry study were captured from the flock at the Bang Poo study site, Samut Prakarn province, Thailand (Figure 1). They were of adult age, negative for influenza virus infection (see Materials and Methods), physiological healthy and energetic. Tagging the birds with satellite transmitters was performed in two consecutive years. The first Brown-headed gull was tagged in March 2008; and the other 7 gulls were tagged between February and March 2009. Flyways of these gulls were monitored until the satellite signals were lost. Demographic data and duration of tracking of each bird are shown in Table 1. Two gulls (I.Ds. 74795 and 88216) completed one migratory cycle, 3 gulls (I.Ds. 88215, 91416 and 91417) completed 2 cycles (Table 2); while 3 gulls (I.Ds. 88217, 88218 and 91418) were lost in Thailand within one month after tagging (Table 1). Flyways of the 5 marked gulls including their geographical locations in different countries are as shown in Table 3, and individual flyway of each gull was described in detail in Supporting Information S1.

**Table 1:** Demographic data of the tracked gulls.

Bird I.D.	Sex	Body weight (g)	Marking date	Time at signal loss from monitoring	Place at signal loss	Tracking period
74795	ND	475	Mar 25, 2008	Dec 2008	Cambodia	9 mos.
88215	Male	650	Mar 26, 2009	Mar 2011	Thailand	2 yrs.
88216	Female	600	Mar 13, 2009	May 2010	Tibet	1 yr, 2 mos.
88217	ND	450	Feb 17, 2009	Mar 2009	Thailand	<1 mo.
88218	Female	500	Mar 26, 2009	Apr 2009	Thailand	<1 mo.
91416	ND	440	Feb 17, 2009	Jan 2011	Cambodia	1 yr, 11 mos.
91417	ND	430	Feb 17, 2009	Nov 2010	Thailand	1 yr, 9 mos.
91418	ND	400	Feb 17, 2009	Mar 2009	Thailand	<1 mo.

ND = Not determined.

**Table 2:** Flying distances of the tracked gulls.

		Total distance on migration (km)		Duration of migration (days)		Average flying distance on migration per day (km)		Average distance at habitat per day (km)	
Bird I.D.	Year	Thailand to China	China to Thailand	Thailand to China	China to Thailand	Thailand to China	China to Thailand	Thailand	China
74795	2008	2,419	2,747	7	9	346	305	28	26
88215	2009	3,167	2,372	12	16	264	148	7	2
	2010	2,223	2,404	9	21	247	114	6	9
	2011	Lost						6	
88216	2009	2,343	2,014	5	15	469	134	6	19
	2010	2,074	Lost	7		296		2	
91416	2009	2,255	2,067	12	12	188	172	2	1
	2010	1,954	1,968	23	12	85	164	3	3
91417	2009	2,924	2,917	39*	22	ND*	133	6	3
	2010	2,403	2,870	22	5	109	574	3	10
Average distance		2,418	2,420	12	14	202	173	7	9
95% CI		2,139–2,698	2,143–2,697	7–23	10–18	165–342	115–311	1–12	3–16

CI = Confidence interval.

* ND = Not determine; because the migratory route started from Thailand to Cambodia and Vietnam, and it took 39 days from Vietnam to China.

**Table 3:** Habitats of the tracked gulls.

		Length of stay (days)				
Bird I.D.	Location	2008	2009	2010	2011	Average
74795	Thailand: Samut Prakan, Samut Sakhon	44	-	-	-	-
	China: Qinghai, Xinjiang, Tibet	174	-	-	-	-
	Cambodia: Siem Reap	35	-	-	-	-
88215	Thailand: Samut Prakan, Samut Songkhram	-	90	119	61	90
	China: Xinjiang, Tibet	-	170	180	-	175
	Cambodia: Pursat, Siem Reap	-	-	40 (02/12/10 -11/01/11)		-
88216	Thailand : SamutPrakan, SamutSongkhram, Chachoengsao	-	90	98	-	94
	China: Tibet	-	183	-	-	-
91416	Thailand :SamutSongkhram	-	64	101	-	82
	China: Tibet	-	201	189	-	195
	Cambodia: Kampong Thom, Pursat, Siem Reap	-	-	42 (19/11/10 - 10/01/11)		-
91417	Thailand : Samut Prakan, Samut Songkhram	-	83	78	-	81
	China: Qinghai, Xinjiang	-	146	180	-	163
	Cambodia: Kampong Thom, Pursat, Siem Reap	-	32 (25/12/09- 25/01/10)		-	-

**Figure 2:**
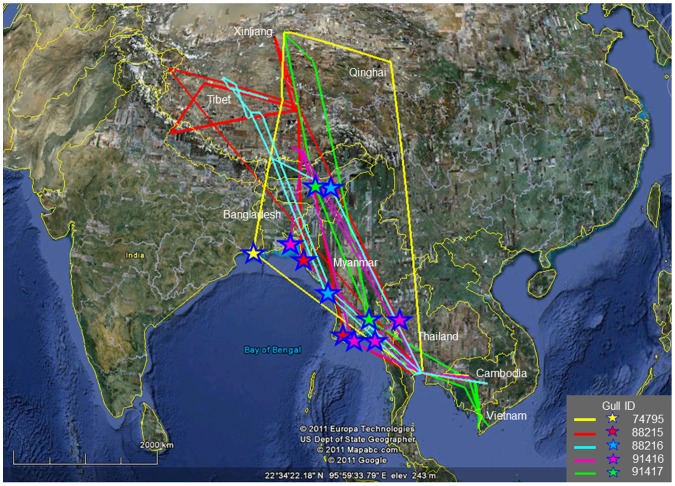
Migratory routes of all study gulls. (A) Gull I.D. 74795; (B) Gull I.D. 88215; (C) Gull I.D. 88216; (D) Gull I.D. 91416; and (E) Gull I.D. 91417. Color stars indicate the stopover places.

The mean flying distance of the tagged gulls during migration from Thailand to China was 202 km/day (165–342 km/day at 95% CI) and it was 173 km/day (115–311 km/day at 95% CI) from China to Thailand; whereas the local movements within a given site was 7 km/day (1–12 km/day at 95% CI) in Thailand and 9 km/day (3–16 km/day at 95% CI) in China (Table 2). In general, gulls traveled an estimated migratory distance of 2,400 km between China and Thailand with duration of migration of approximately 12 days in average (7–23 days at 95% CI). Although all gulls began their flight from the same study site in Thailand, their destinations in China were different. They also arrived and left Thailand at different time points. These results indicated that these Brown-headed gulls belong to different flocks while they were in China, and just gathered together in the same overwintering site in Thailand. Migratory routes of the 5 tracked gulls spanned 7 countries- Thailand, Myanmar, India, China, Bangladesh, Vietnam and Cambodia (Figure 2). Almost all tracked gulls, except gull I.D. 88216, stayed in Cambodia for approximately 30–40 days in a migratory season (Table 3); and only the gull I.D. 91417 had traveled further to Vietnam. If seasonal habitat is defined as the places where gulls stayed for longer than 1 month, those places were mainly located in three countries: China, Thailand and Cambodia; while Myanmar, India, Bangladesh and Vietnam were considered to be the stopover countries. Moreover, the stopover places of individual gull in each country were different. They could be West Bengal or Assum in India, the Gulf of Martaban, Ayeyarwaddy or Rakhine in Myanmar and Kien Giang in Vietnam. In the three gulls that were tracked for over two migratory cycles, the migratory routes were only slightly different between years.

**Figure 3:**
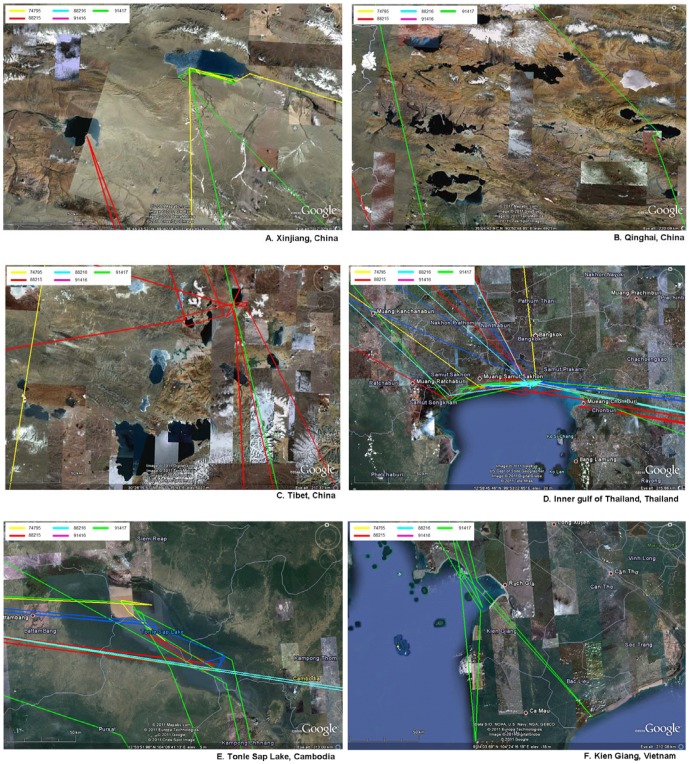
Ecological characteristics of habitats in each country involving the inland lakes and coastal areas.

**Figure 4:**
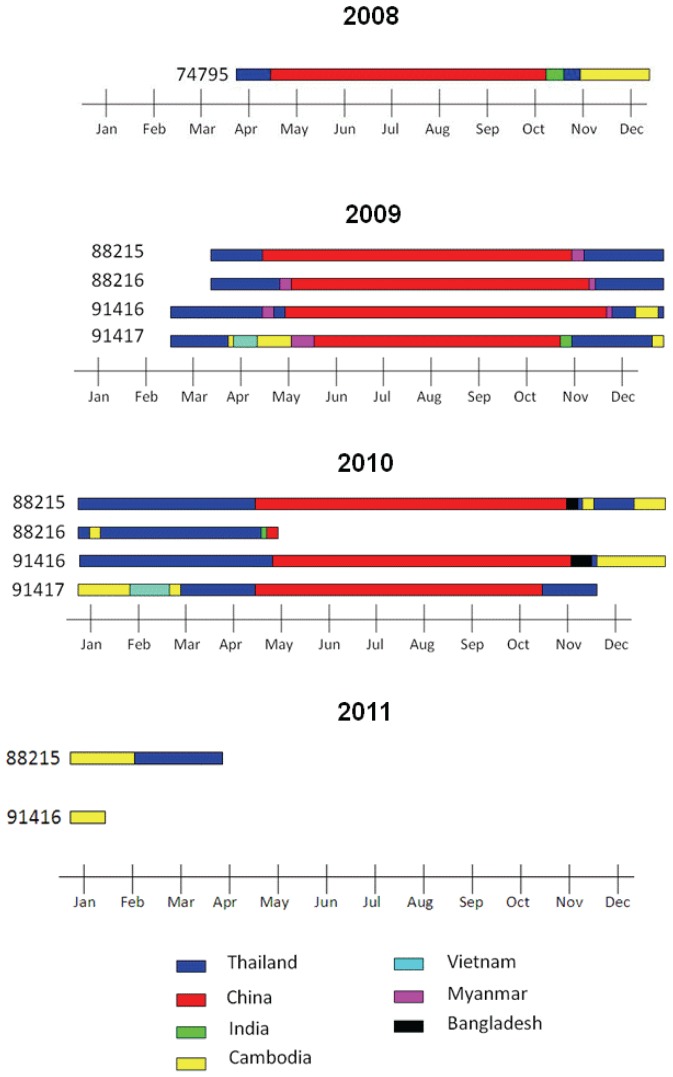
Duration of stay of each gull in various countries.

Ecologically, seasonal habitats were always along the coast, wetlands or inland lakes (Figure 3). Birds stayed over the cold season for 5–6 months in Thailand and stayed in their breeding places in China for another 5–6 months. Duration that gulls stayed in each country is shown in Figure 4. Seasonal habitats in Thailand were mainly in three provinces: Samut Prakan, Samut Sakhon and Samut Songkhram, all of which were situated along the inner gulf of Thailand. In China, the breeding habitats were in Tibet, Qinghai and Xinjiang. However, gulls also spent their times at a third habitat in Siem Reap, Cambodia around Tonle Sap, the largest fresh water lake in Southeast Asia [23]. Although this flock of gulls stayed through winter along the inner gulf of Thailand, no AIV outbreak was reported in that area through the extensive national surveillance system.

### Prevalence of avian influenza virus infection in gulls

A total of 75 birds were captured from the Bang Poo study site during January to March of 2008, 2009 and 2010. Throat and cloacal swabs as well as blood samples were collected for the investigation of influenza virus infection. No influenza virus was isolated and the viral genome was not detected in cloacal and throat swab samples. The H5N1 antibody was not detected in the hemagglutination-inhibition (HI) and microneutralization (MicroNT) assays. However, 6.6% of samples were positive for the pan-influenza A antibody by ELISA.

### Tolerability of gulls to HPAI H5N1 virus infection

Brown-headed gulls kept in captivity were inoculated with HPAI H5N1virus in order to explore whether the infected birds could survive the infection, and therefore might be able to carry on migratory activity. Group of 3 gulls was inoculated with the H5N1 virus at the inoculum dose of 10, 10^3^ or 10^4^ tissue culture infective dose 50 (TCID50) per head (Table 4). The result demonstrated that with the high inoculum dose of 10^3^ or 10^4^ TCID50 all inoculated gulls began shedding the virus intermittently in cloacae and trachea after 1–2 days post- inoculation (dpi.). As determined by RT-PCR, titers of the viruses shed from trachea from the first day of detection to the day of death were at range of 10^5^ to 10^6^ copies/ml or undetectable; and the tracheal specimens were also positive for virus isolation in Madin Darby canine kidney (MDCK) cell cultures (Table 4). The virus shed from cloacae was detected by the isolation method only, but not by RT-PCR with sensitivity of detection of 98 copies/reaction. The birds inoculated with these two inoculums doses could tolerate the infection and died between day 4 and day 6 post-inoculation. At one day before death, apparent clinical symptoms such as ataxia, ventral recumbence, depression, ruffled feather and shaking head were noticed. Regarding the inoculums dose of 10 TCID50, 1 of 3 inoculated gulls could shed the virus from cloacal sample only at 3 dpi. as determined by isolation method only. On day 4, virus shedding was also detected from tracheal sample by isolation method only. This bird showed symptom of ataxia on day 6 and died on day 7. Necropsy of this bird showed brain congestion and ecchymatic hemorrhage in gizzard. The two gulls that survived the infection neither shed the virus nor developed any clinical symptom, of which one of them underwent seroconversion.

**Table 4:** Susceptibility of Brown-headed gulls in captivity to H5N1 virus challenge.

		Trachea			Cloacae				NT antibody titers at day:					
Inoculum dose (TCID50)	Gull I.D.	Day of shedding p.i.	Viral titer (copies/ml)	Isolation	Day of shedding p.i.	Viral titer (copies/ml)	Isolation	Day of death	0	5	10	15	20	25
10^4^	1	Not shed	-	-	2	und	+	4	<20	ND	ND	ND	ND	ND
					3	und	+							
	2	2	und	+	2	und	+	4	<20	ND	ND	ND	ND	ND
		3	und	+										
	3	1	1.18×10^6^	+	3	und	+	5	<20	ND	ND	ND	ND	ND
		2	und	+	4	und	+							
		3	und	+										
		4	und	+										
10^3^	4	3	1.62×10^5^	+	Not shed	-	-	5	<20	ND	ND	ND	ND	ND
		4	und	+										
	5	1	und	+	2	und	+	5	<20	ND	ND	ND	ND	ND
		2	2.08×10^5^	+	3	und	+							
		3	2.78×10^5^	+	4	und	+							
		4	und	+										
	6	2	5.68×10^5^	+	3	und	+	6	<20	20	ND	ND	ND	ND
		4	und	+										
10	7	Not shed	-	-	Not shed	-	-	No	<20	<20	<20	<20	<20	<20
	8	Not shed	-	-	Not shed	-	-	No	<20	<20	160	1280	ND	ND
	9	4	und	+	3	und	+	7	<20	<20	ND	ND	ND	ND

und = undetectable, p.i. = post inoculation.

Note: Sensitivity of RT-PCR is 98 copies/reaction.

## Discussion

The worldwide resurgence of H5N1 HPAI since 2003 has resulted in massive deaths in poultry and human deaths, especially in East and Southeast Asian countries. Integrated data and phylogenetic analyses of virus isolates obtained from different geographical locations suggest that the H5N1 spread throughout Asian and African countries involved both migratory birds and trade in poultry, whereas the spread in European countries involved migratory birds [24]. Human population, duck population (particularly, free grazing ducks) and intensity of the rice crop can increase the risk of propagation of H5N1 HPAI in Southeast Asian countries [25], [26]. There is a close relationship between the density of free grazing ducks and the double or triple cultivation of rice in Thailand, because the falling rice grains left in the fields after harvesting are the source of low-cost food for ducks. Movements of free grazing ducks to post-harvested rice fields has been suggested as the major source of virus spread in Thailand during the initial waves of HPAI outbreaks. Nevertheless, duck movement has been later on prohibited. Natural infection in the other animal species such as tigers and leopards, cats, and dog was mainly caused by ingestion of infected carcasses or chicken meat [27]–[30]. However, most cases of H5N1 HPAI infection in humans in Thailand were mainly caused by exposure to sick or dead chicken.

It has been very difficult to trace back how the H5N1 HPAI virus was introduced into Thailand because of the delay between the initial cases and the subsequent spread of the virus throughout of the country [31]. It has been hypothesized that the virus might have been introduced via migratory birds. HPAI usually occurs during low temperature months (October to February) which coincide with the time of arrival of wintering migratory birds [31]. In our study, Brown-headed gulls seem to be a prime candidate species for spreading avian influenza. They are long distant migrant and their flyways involved 7 countries, all of which had been affected by the H5N1 HPAI. Nevertheless, on the basis of an inhabiting time of 1 month or longer, only China, Thailand and Cambodia, were considered to be the gulls' seasonal habitats, whereas Myanmar, Bangladesh, India and Vietnam were stopover places. In our study, Brown-headed gulls spent their breeding time in the lake areas of Tibet, Qinghai and Xinjiang. All of these breeding sites are cold and high altitude lakes of varying salinity [21]. They migrated to warmer places and stayed overwinter in mudflats along the inner gulf of Thailand and Tonle Sap Lake areas in Cambodia.

Our study illustrates complete migratory cycles of Brown-headed gulls. Gulls in this flock arrived and left Thailand asynchronously; their destinations on migration to China were different, suggesting that they belonged to different flocks while in China. Gulls from these flocks gathered together during the winter time in Thailand. The first group of gulls reached the Bang Poo study site in October and the last group left in May. The migration distance of about 2400 km between Thailand and China and the migration time at range of 5–23 days in our tracked Brown-headed gulls were more or less similar to those previously reported in wildfowl in which the movements of up to 2,900 km in 5–15 days were within the timeframe that was compatible with the preclinical symptom and virus dispersal [16].

We looked for a linkage between the virus from the Qinghai outbreaks and the H5N1 HPAI viruses isolated from Brown-headed gulls in Thailand in 2005 and 2008, but no linkage was found. Phylogenetic analysis showed that the viruses isolated from birds in Qinghai in 2005 belonged to clade 2.2 [10], [11], whereas the viruses isolated from gulls in Thailand between 2005 and 2008 belonged to clade 1 (unpublished data). Nevertheless, phylogenetic analysis showed a virological linkage between countries situated in the migratory corridor of this flock of Brown-headed gulls. The viruses causing outbreaks in Bangladesh belonged to the Qinghai like-lineage [32]; and the viruses causing the first epidemic wave in Thailand, Cambodia and Vietnam belonged to clade 1 [8], [33]. The clade 1 virus was originally isolated in Yunnan, southern China in 2002 and 2003, and probably spread to Vietnam by poultry trade across the shared border that is 600 km long [34]. Thereafter, the viruses spread from the North to the South of Vietnam. Interestingly, the H5N1 virus isolates in Thailand are closely related to the isolates in Vietnam [6]. It is also claimed that the clade 1 virus spread from Thailand to Cambodia [34]. Nevertheless, it remains unknown for how the clade 1 virus was introduced into Thailand. The firstly H5N1 HPAI outbreaks in Thailand occurred in central part, not at the border of the country. Interestingly, Vietnam, Thailand and Cambodia reported the first outbreak in the country at about the same period of time in 2004, i.e., on January 23 for Thailand, on January 8 for Vietnam, and January 24 for Cambodia [6]. Although there might be a lag time period for outbreak identification and report, it is implied that the H5N1 HPAI outbreaks occurred in these countries more or less about the same time. In addition, the time at occurrence of outbreaks in these 3 countries coincides with the overwintering period of Brown-headed gulls.

Our study showed that Brown-headed gulls kept in captivity were vulnerable to HPAI H5N1 virus. Nevertheless, gulls infected with the inoculum dose of 10^4^ TCID50 could survive for 4–5 days and those inoculated with 10^3^ TCID50 could survive for 5–6 days, and with viral shedding from trachea and cloacae which began after 1 dpi. In addition, a gull infected with the low inoculum dose of 10 TCID50 could survive for 7 days with viral shedding which began at 3 dpi. Apparent clinical symptoms were noticed at just one day before death indicating that the infected gulls may be able to complete migratory route between China and Thailand, or at least, at a shorter distant between nearby countries. Gulls inhabited and stopover in water rich areas where numerous domestic and migratory bird species can be found [22], [35], [36]; therefore, the infected gulls might be able to spread the virus to the other birds foraging in the same vicinity.

Although cross border trade may be the major route of H5N1 HPAI virus spread in Asian countries, it cannot be excluded that migratory birds might also play an additional role in the spread of the virus across countries or domestically along its flyways. In spite of the fact that Brown-headed gulls appear to be an ideal candidate for spreading the virus, no H5N1 virus including other influenza subtypes could be isolated, and no H5N1 antibody could be detected in our flock of Brown-headed gulls during the study period. Only anti-influenza antibody was detected by ELISA, indicating previous infection with some subtype of AIVs in these birds. Besides waterfowl, gulls and shorebirds also maintain an influenza gene pool in their species. Predominant AIV subtypes found in gulls are H13 and H16 [2].

The inability to detect the H5N1 virus, however, may simply be due to our small sample size collected from gulls as all migratory birds in Thailand are protected by law; and it may be also due to the absence of HPAI outbreaks during our investigation period of 2008–2011. There was only one outbreak occurred in poultry in Nakhon Sawan and Phichit provinces (upper part of central Thailand) in January 2008, and one in backyard poultry in Sukhothai province (lower north of the country in November 2008). No AI outbreak has been reported since then [6]. Satellite telemetry was conducted in Bar headed geese and Ruddy Shelducks in Qinghai Lake, China and also in Chilika Lagoon and Khunthankulum Reserves, India in order to determine correlation between flying route and HPAI H5N1 outbreak in South Asia [37]. Nevertheless, this study is the first flyway monitoring of the Brown-headed gulls that covered complete migratory seasons and encompassed the epicenter of H5N1 HPAI in Southeast Asia which has never been reported before.

## Materials and Methods

### Approvals and permissions

This study was approved by the Animal Care and Use Committee of the Faculty of Veterinary Science, Mahidol University, and compiled with the Statement of Compliance (Assurance) with Standards for Humane Care and Use of Laboratory Animals of the Office of Laboratory Animal Welfare (OLAW No. A5731-01), U.S. Department of Health and Human Services. The study was carried out with permission of the Department of National Parks, Wildlife and Plant Conservation, Thailand, in accordance with the Wildlife Conservation Act of Thailand.

### Study site

This study was conducted at Bang Poo, a recreation area located in the Samut Prakan province, Thailand, 37 km east of Bangkok. This 63 km^2^ coastal area owned by the Royal Thai Army is a part of the inner gulf of Thailand and comprised mangrove and large mudflat habitats. Gulls gather together here to feed on an abundance number of clams that embedded in the muddy beaches. Bang Poo is an Important Bird Areas (IBA) with more than 135 water bird species being recorded, including 7 species of ducks, 50 species of waders, and 18 species of terns and gulls. Thirteen globally threatened species have been recorded in this area [22]. The Brown-headed gull is an important species in this wetland IBA [19]–[22].

### Study birds

The Brown-headed gull (*Larus brunnicephalus*) is a waterfowl belonging to the family *Laridae*. The common name for this species comes from the presence of brown color on the head of adult birds. These gulls normally feed on small fishes and crabs; nevertheless, they can adapt to a variety of available foods.

### Bird capture and specimen collection

All migratory birds in Thailand are protected animals by law. Therefore, bird capture was performed by the authorized persons from the Department of National Park, Wildlife and Plant Conservation. Gulls were attracted by fried pork rind, and captured by a hand net. Approximately 10–20 birds were captured in each trap. Oropharyngeal/tracheal and cloacal swabs as well as blood samples were collected for laboratory investigations to determine the prevalence of AIV infection. The captured birds were also physically examined for general health conditions (body weight, body size) and the healthy, energetic adults that were negative for influenza viral infection, as screened by rapid antigen detection kit (Rapid H5 AIV Ag Test, Bionote, Inc., Gyeonggi-Do, South Korea) at the study site, were chosen for satellite telemetry tracking. Results of samples negative for the virus infection by antigen detection were confirmed at the Virology Laboratory at the Faculty of Veterinary Science, Mahidol University.

### Satellite telemetry technique and data analysis

Eight gulls negative for influenza antigen were chosen for the satellite telemetry study. The first gull was marked in March 2008 and the second lot of 7 gulls was marked during February and March 2009. Each bird was tagged with a ring band and fitted with a solar powered satellite platform transmitters (solar PTT-100, Microwave Telemetry, Inc., Columbia, MD) on its back by using Teflon harnesses (Bally Ribbon Mills, Bally, PA). A transmitter weighing 12 g was used for the first gull and transmitters weighing 9.5 g were used for remaining 7 gulls. On average, the transmitter packages weighed approximately 1.98% of the bird's body weight. After being marked, birds were released, usually within 1 hour, to a place close to the capture sites.

The solar PTT-100 transmitter operates at frequency of 401.650 MHz. The standard duty cycle of the solar PTTs was set at 10 hours on and 48 off for recharging the batteries. Signals were processed and the data was provided by Argos CLS (Toulouse, France). “Bird locations were analyzed at location classes 1, 2 or 3 only and mapped with Google Earth Program version 5.1 (Google, Mountain View, CA, USA); and a precision of less than 1500 meters error was obtained.”

When the transmitter signal from any tracked bird was lost, the observation was still going on for at least one more month before concluding its disappearance.

### Detection of H5N1 HPAI infection in study birds

Prevalence of influenza virus in this flock of gulls as well as the confirmation of the negative results for influenza antigen detection was determined by real time reverse transcription-polymerase chain reaction (RT-PCR) for viral genome detection and the virus isolation from throat and cloacal swab samples. Serological techniques for detection of H5 antibody were performed by HI and microNT assays; and that for detection of pan-influenza antibody was performed by ELISA.

#### Real time RT-PCR

Protocols from the Organization des Epizooties (http://www.oie.int/fr/normes/mmanual/2008/pdf/2.03.04_AI.pdf) and/or those established by the U.S. Centers for Disease Control and Prevention (CDC) were used for viral genome detection and subtype identification. Throat and cloacal swabs from each bird were tested separately.

#### Virus isolation method

Cloacal and throat swab specimens were separately inoculated in duplicate in embryonated chicken eggs and MDCK cell monolayer. Amniotic/allantoic fluid and culture supernatant were screened for presence of influenza virus by hemagglutination with 0.5% goose red blood cells before subjected to subtype identification by real time RT-PCR.

#### HI assay

The protocol for H5N1 antibody detection was based on the method given for avian influenza in the World Health Organization (WHO) manual on animal influenza diagnosis and surveillance [38] in which 0.5% goose erythrocytes were chosen as the indicator as previously described by Louisirirotchanakul et al. [39]. The assay was performed in micro-titer V plate in duplicate wells using replicating A/chicken/Thailand/ICRC-V143/07(H5N1) (accession No. EU233413–EU233420) at a final concentration of 4 hemagglutination units/25 µl as the test antigen. Titer was defined as the highest serum dilution that causes complete hemagglutination of the test erythrocytes.

#### microNT assay

H5N1 antibody was detected by ELISA based microNT assay using the protocols as described in the WHO manual for avian influenza [38], and modified by Louisirirotchanakul et al. [39] in which MDCK cell suspension was replaced with MDCK cell monolayer. The assay was performed in micro-titer plates in duplicate. A/chicken/Thailand/ICRC-V143/07(H5N1) at a final concentration of 100 TCID50 was used as the test virus. Viral nucleoprotein synthesized was detected by a mouse monoclonal antibody (Chemicon International, Inc., Tecumala, CA) as the primary antibody together with horse radish peroxidase-conjugated rabbit anti-mouse Ig (Dako Cytomation, Denmark) as the secondary antibody. Titer was defined as the highest serum dilution that causes a 50% reduction in the amount of viral nucleoprotein synthesized.

#### ELISA

Sera were assayed for antibody to influenza A viruses (pan-influenza A subtypes), using the type A influenza multi species antibody test kit (AI MSp) (BioChek, London, UK) according to the manufacturer's instructions.

### Experimental HPAI H5N1 virus infection

To study whether infected birds could survive the infection and still be able to carry on migratory activity, wild birds were trapped and kept in captivity for a week prior to viral inoculation. These gulls were negative for influenza virus infection as tested by RT-PCR and virus isolation method. A/Brown-head gull/Thailand/VSMU-28-SPK/2005(H5N1) which was isolated from an apparently healthy looking gull in the surveillance study of HPAI H5N1 virus infection in wildlife birds was used as the challenging virus. Each bird was intranasal inoculated with a 100 µl volume of an inoculum dose of 10, 10^3^ or 10^4^ TCID50. Inoculated birds were kept in an isolator in an animal biosafety laboratory level 3, Faculty of Veterinary Science, Mahidol University. Tracheal and cloacal swab specimens were collected daily till death. Each swab sample was kept in 2 ml of viral transport media and kept frozen at −80 C until tested. Birds were also observed daily for signs, symptoms and death until one month after inoculation.

## Supporting Information

Supporting Information S1 **Individual flyway of each 5 marked gull was described in details in supporting information.**
(DOCX)
