# Supplementary material for: Satellite Tracking on the Flyways of Brown-Headed Gulls and Their Potential Role in the Spread of Highly Pathogenic Avian Influenza H5N1 Virus
Source: PLoS One. 2012 Nov 28;7(11):e49939. doi: 10.1371/journal.pone.0049939 (PMC3509151; doi:10.1371/journal.pone.0049939)
Supplement: Supporting Information S1 — Individual flyway of each 5 marked gull was described in details in supporting information. (DOCX) [file pone.0049939.s001.docx]

Supporting information

*Gull I.D. 74795* was the first gull to be tagged in our study. It was tagged on March 25, 2008 and departed Thailand on April 4, 2008. It spent about 1 week to cover an average distance of 2,419 km to reach Qinghai in western China. This gull inhabited three areas in China (Qinghai, Xinjiang, and Tibet) for 174 days; and then left Tibet for Thailand on October 11, 2008. On the way back to Thailand, this gull stopped over in West Bengal, India for refueling before moving southeast and arriving at Samut Sakhon province, Thailand on October 20, 2008. In total, this bird spent approximately 9 days on its return trip, and covered a distance of 2,747 km. After staying in Thailand for approximately 1 week, this gull moved further to Siem Reap in Cambodia, spending 3 days traveling to reach there on October 30. The signal of this gull was lost in Cambodia on December 4, 2008. Overall, the gull was tracked for 254 days.

*Gull I.D. 88215* was marked on March 26, 2009, approximately one year after the first gull. It left Thailand in mid-April 2009 and spent 12 days with a flying distance of about 3,167 km to reach Aksai Chin before moving on to Tibet, China. The gull stayed in Tibet for 170 days before heading back in late October 2009 to Thailand to overwinter. The bird stopped over at Ayeyarwaddy, Myanmar and arrived Samut Songkhram province, Thailand in early November. Totally, this gull spent approximately16 days travelling from Tibet to Thailand, and covered an average distance of 2,372 km. The gull wandered around the inner gulf of Thailand for 158 days, and then left for Tibet again in April 2010 to complete the first migratory cycle. The gull left Tibet for the second time in October 2010 with a stopover in Bangladesh. During the overwintering period, this gull flew back and forth between Thailand and Cambodia; and finally was lost from our tracking while in Thailand in late March 2011. Totally, this gull was tracked for 2 years.

*Gull I.D. 88216* was marked on March 13, 2009. Subsequently, it left Thailand, stopped over in Rakhine, Myanmar, and reached Tibet in late April. On this migration, the gull traveled for about 5 days, covering a distance of 2,343 km. The gull spent its breeding season in Tibet for 183 days, before heading back and arriving Thailand in November, 2009. On its return trip to Thailand, the gull refueled at Rakhine, Myanmar, nearly the same area as it ever used in April. On the return route, the gull spent approximately 15 days covering a distance of 2,014 km to arrive in Samut Songkhram province, Thailand on November 11, 2009. It roamed around this area for 50 days before traveling further for two days to the Tonle Sap Lake area in Kampong Cham, Cambodia. However, it returned to Thailand after staying in Cambodia for only a few days, and spent most of its wintering time in Thailand (approximately 98 days). After that, the gull headed to Tibet with one stopover in Assum, India. Totally, this gull spent approximately 7 days (including stopover) covering a distance of 2,074 km on the way to Tibet. Finally, the signal of this gull was lost in Tibet in May 2010, after the gull had been tracked for 14 months.

*Gull I.D. 91416* was marked on February 17, 2009. After marking, the bird roamed around the inner gulf of Thailand for approximately 2 months before leaving for Tibet in late April. On the migratory route, the gull had two stopovers at the Gulf of Martaban, Myanmar and in Tak province, Thailand. The gull spent approximately 12 days, including the stopover period, and covered a distance of 2,255 km. This gull stayed in Tibet for 201 days during the breeding season, and left Tibet in November to return to Thailand with stopovers at Ayeyarwaddy and the Gulf of Martaban, Myanmar. The gull traveled for 12 days, including the stopover period, on this migratory route, covering a distance of 2,067 km and arrived in SamutPrakan province, Thailand on November 26, 2009. This gull moved around this area for 7 days and then traveled further to the Tonle Sap Lake area in Pursat, Cambodia and stayed there for 17 days before returning to Thailand again. The gull stayed along the inner Gulf of Thailand for more than 3 months before departing to Tibet in April, 2010, and reached its destination in 23 days, covering a total distance of 1,954 km. The gull was in Tibet for approximately 6 months, and then flew to Bangladesh in early November and returned to Thailand in mid-November, 2010, covering a distance of 1968.2 km. However, the gull moved on to Siem Reap, Cambodia and the satellite signal was lost at the Tonle Sap Lake area in January 2011. This gull was tracked for 1 year and 11 months.

*Gull I.D. 91417* was marked on the same day as gull ID 91416 (February 17, 2009), but it migrated out of Thailand earlier. It left Thailand on March 26, 2009 heading to the Tonle Sap Lake area in Pursat, Cambodia and then traveling further to KienGiang, Vietnam. The gull roamed around that area for about 10 days and migrated further to Qinghai, China with stopovers at Kampong Chhnang and Kampong Thom, Cambodia and Mawlamyine, Myanmar. On the migratory route from Vietnam to China, this gull spent 39 days, covering a distance of 3,196 km. The gull wandered around Qinghai and Xinjiang, China during the breeding season for almost 5 months. It then migrated back to Thailand in October, 2009 with stopovers in Assam, India and Mawlamyine, Myanmar. The gull arrived in Samut Songkhram province, Thailand on November 1, 2009 after traveling for 22 days (including the stopover time), covering a distance of about 2,917 km.

After roaming around the inner gulf of Thailand for 46 days, the bird flew further to the Tonle Sap Lake area, Cambodia and stayed there for 32 days. The gull then moved further to the coast of Kien Giang, Vietnam and stayed there until late February before returning to Samut Prakan province, Thailand, in late February, 2010. The gull left Thailand for China in April 2010 and inhabited the Qinghai and Xinjiang areas for 180 days. The gull migrated back to Thailand again in mid-October and its signal was lost in November 2010. The total tracking period of this gull was 1 year and 9 months. This gull had the longest flyways among all birds studied (3,517 km difference between the most northern and the most southern areas). The flyway of this gull is shown in Figure 2.
